# Central Venous Oxygen Saturation as a Predictor of a Successful Spontaneous Breathing Trial from Mechanical Ventilation: A Prospective, Nested Case-Control Study

**DOI:** 10.2174/1874306401812010011

**Published:** 2018-03-26

**Authors:** Ioannis Georgakas, Afroditi K. Boutou, Georgia Pitsiou, Ioannis Kioumis, Milly Bitzani, Kristina Matei, Paraskevi Argyropoulou, Ioannis Stanopoulos

**Affiliations:** 1 Respiratory Failure Unit, Aristotle University of Thessaloniki, Thessaloniki, Greece; 2 Department of Respiratory Medicine, “G. Papanikolaou” Hospital, Thessaloniki, Greece; 3 1st Intensive Care Unit, “G. Papanikolaou” Hospital, Thessaloniki, Greece; 4 Pulmonary Department, Aristotle University of Thessaloniki, Greece; 5 Intensive Care Unit, General Hospital of Veroia, Veroia, Greece

**Keywords:** Central venous oxygen saturation, Spontaneous breathing trial, Weaning, Mechanical ventilation, Nested case-control study, Oxygen extraction ratio

## Abstract

**Background:**

Weaning from mechanical ventilation is a key element in the care of critically ill patients, and Spontaneous Breathing Trial (SBT) is a crucial step in this procedure. This nested case-control study aimed to evaluate whether central oxygen saturation (ScvO_2_) values and their changes could independently predict the SBT outcome among mechanically ventilated patients.

**Methods:**

A prospective cohort of patients who were mechanically ventilated for at least 48hours and fulfilled the criteria of readiness to wean constituted the study population. All patients attempted a SBT and were then categorized in SBT success group and SBT failure group, based on a combination of criteria which indicated whether SBT was successful or not. Multivariate binary logistic regression analysis was utilized to indicate the independent predictors of SBT success, while the Receiver Operating Characteristic (ROC) curves were used to demonstrate the diagnostic accuracy of these independent predictors.

**Results:**

Seventy-seven patients 69(18-86) years old; 62.3% male) constituted the study population. SBT was successful among 63.6% of them. A decrease in ScvO_2_ values (ΔScvO_2_) < 4% between the beginning and the end of the trial independently predicted the successful outcome (OR=18.278; 95% CI=4.017-83.163), along with age, Hemoglobin concentration (Hb) and arterial oxygen saturation (SaO_2_). Diagnostic accuracy for ΔScvO_2_ alone (ROC area=0.715) was slightly superior to that of either SaO_2_ (0.625) or Hb (0.685) to predict SBT success.

**Conclusion:**

ScvO_2_ is an independent predictor of the weaning outcome and its evaluation may further facilitate the accurate categorization among those patients who pass or fail the SBT.

## 1 INTRODUCTION

Weaning patients from mechanical ventilation are one of the key elements in the course of the treatment of critically ill patients and could represent up to 40% of the time that a patient spends on mechanical ventilation [1, 2]. A prolongation of mechanical weaning is associated with increased risk of complications, such as ventilator-associated pneumonia, tracheal injury, bleeding from digestive stress ulcers and sepsis [3-5], while early extubation and re-intubation could result to nosocomial pneumonia and increased mortality [6, 7]. Thus, identifying the optimal point when a patient can sustain spontaneous breathing is essential, but could be challenging.

In order to facilitate physicians in identifying the patients who are ready to wean from mechanical ventilation after the resolution of acute disease phase, a number of criteria have been set, in a two-step procedure [8], which begins with a trial of spontaneous breathing (SBT) *via *a T-tube. Since no single and consistent predictor has been found to accurately predict the SBT outcome [9, 10], the decision of a successful or not trial is based on a combination of another set of criteria evaluating the hemodynamic or not stability of the patient and the change of mental status, oxygenation and ventilatory efficiency during spontaneous breathing [8]. Previous data indicate that global tissue oxygenation is an important predictor of the weaning outcome [11, 12] and is associated, especially among patients who fail to wean, with the increased oxygen cost of breathing due to the strenuous working respiratory muscles [12]. Mixed venous oxygen saturation (SvO_2_), which requires invasive measurements *via *right heart catheterization, and the easier to obtain central venous oxygen saturation (ScvO_2_), are two indices closely associated with global tissue oxygenation; Nevertheless, their routine screening is currently not recommended as part of the evaluation of the SBT outcome. Although SvO2 and ScvO2 measurements have been previously studied in regards to several outcomes and in several patient populations [2, 13, 14], data on the prognostic accuracy of SvO_2_ values, ScvO_2_ values and their changes on the weaning outcome, is currently limited [2, 15, 16]. Moreover, most of these studies were conducted in selected patient populations [2, 15] or aimed to investigate whether ScvO_2_ and SvO_2_ could predict the extubation outcome, which is the final and not the initial step of the weaning procedure [2, 16] and could be, thus, influence by several other factors not closely associated with tissue oxygenation, such as an inadequate cough or laryngeal edema [10].

To the author’s knowledge, data on the clinical significance of ScvO_2_ as a potential predictor of the SBT outcome is currently scarce. Thus, we conducted a prospective study aiming to evaluate whether ScvO_2_ values and their changes between the initiation and the end of a thirty-minute long SBT could independently predict the outcome of this trial, in a general surgical and medical population of mechanically ventilated patients.

## 2 MATERIAL AND METHODS

### 2.1 Study Population

This is a prospective nested case-control study. All patients who were under mechanical ventilation for at least 48 hours in two medical Intensive Care Units (ICU) of “G. Papanikolaou” hospital between January 2012 and December 2012, were eligible for the study. Patients who were intubated for less than 48 hours, who had undergone an early tracheostomy (in less than 48 hours), who died before any weaning trials from mechanical ventilation were attempted and/or who did not have a central venous catheter, were excluded from final analysis; the rest constituted the final study population. All patients underwent a 30-minute long SBT before an attempt for extubation was undertaken and based on the outcome of the trial were then separated in two groups: the group of patients with successful SBT (cases) and the group of patients with SBT failure (controls). The Research Ethics Committee of the Aristotle University of Thessaloniki approved the study protocol and informed consent was taken either by the patients or their first-degree relatives.

### 2.2 Study Measurements

Demographic and anthropometric characteristics (gender, age, height, and weight), Acute Physiology and Chronic Health Evaluation (APACHE II) score at first 24hours of ICU stay, days on mechanical ventilation until the first weaning trial was conducted and the ICU admission diagnosis were recorded for all patients. Hemoglobin (Hb) and serum sodium (Na), potassium (K) and calcium (Ca) concentrations were recorded just before the SBT, while arterial blood gases values (arterial oxygen partial pressure (PaO_2_), arterial carbon dioxide partial pressure (PaCO_2_),pH and HCO_3_), arterial oxygen saturation (SaO_2_), ScvO_2_, respiratory frequency (f), Heart Rate (HR) and Systemic Systolic (SBP) and diastolic (DBP) blood pressure were measured at two time points: just before and after the 30 minute long SBT. The Oxygen Extraction Ratio (O_2_ER) was calculated by the equation: O_2_ER=(SaO_2_-ScvO_2_)/SaO_2_ and was also recorded just before the beginning and at the end of the SBT. Moreover, the Rapid Shallow Breathing Index (RSBI), that is the respiratory frequency (f) to Tidal Volume (Vt) ratio (f/Vt) was measured with a handheld Wright spirometer which was placed on the patient’s endotracheal tube at the end of it (at 30 minutes), while the patient’s Maximum Inspiratory Pressure (MIP) was also measured at the end of the SBT, using a manometer with a unidirectional valve.

### 2.3 Study Procedures

All patients were ventilated on Servo I, Servo 300 or Servo 900c (Siemens) ventilators and had a subclavian, femoral or an internal jugular central venous catheter placed; No patient had a Swan-Ganz catheter since its placement is not done during routine clinical practice. Every 24 hours all participants were assessed for readiness to wean by two intensivists, who decided to proceed on a 30 minute long SBT, based on published guidelines [8]. Criteria for readiness to wean included: 1) resolution of acute disease phase which resulted to intubation and mechanical ventilation, 2) adequate cough with not excessive bronchial secretions, 3) hemodynamic stability (no signs of myocardial ischemia, stable SBP (90-160 mmHg) with no or minimal vasoactive drugs (*e.g. *dopamine or dobutamine dosage≤5μg/kg/min) and HR≤140 beats/min), 4) adequate oxygenation (PaO_2_≥60mmHg with Fraction of Inspired Oxygen [FiO_2_]≤40% or PaO_2_/FiO_2_>150 mmHg; Positive End Expiratory Pressure [PEEP]≤8cmH_2_O; and normal (or close to patient’s baseline) PaCO_2_), 5) adequate pulmonary function (MIP≤ -20 cm H_2_O and f ≤35 breaths/min), 6) no major electrolyte disorders and 7) adequate mentation (Glasgow Coma Scale≥13).

All patients who were found to be ready to wean were put on a T-piece with adequate oxygen flow in order FiO_2_ to be similar to that given during mechanical ventilation; the SBT lasted for 30 minutes. Study measurements which were conducted just before the SBT (including arterial and venous blood sampling), were completed while the patients were still on Pressure Support Mode of mechanical ventilation, while those which were conducted at the end of SBT were recorded after 30 minutes of spontaneous breathing on a T-piece.

The SBT was considered as a success or failure at 30 minutes, based on the Task Force criteria, that is both subjective indices and objective measurements. Failure criteria of SBT included: 1) agitation and anxiety, altered mental status, dyspnea, cyanosis and clinical evidence of increased respiratory effort, 2) hypoxemia and/or hypercapnia (PaO_2_<60 mmHg or SaO_2_<90% on FiO_2_≥0.5; PaCO_2_>50 mmHg or ΔPaCO_2_>8 mmHg; pH<7.32 or ΔpH>0.07), 3) ineffective respiratory function (RSBI>105 breaths*min/L), and 4) hemodynamic complications (cardiac arrhythmias, tachycardia [HR>140 beats/min or ΔHR≥20%], hypertension [SBP>180 mmHg or ΔSBP≥20%] or hypotension [SBP<90 mmHg]) [8]. All patients who fulfilled the criteria of SBT success were then extubated.

### 2.4 Statistical Analysis

Statistical analysis was performed using the Predictive Analytics Software (PASW, SPSS Inc) Version 18. The Shapiro-Wilk test of normality was utilized to assess whether variables were normally distributed. Data are presented as mean±1 SD, median (minimum-maximum), or as % percentages based on the normality or not of their distribution. Comparisons between cases and controls were conducted using the Chi-Square test for qualitative variables, while for quantitative variables either the Independent Samples Student’s t-test or the Mann Whitney-U test was utilized, based on the distribution of their values. All variables which were found to be univariately associated with a successful SBT outcome were then entered into a multivariate stepwise binary logistic regression analysis model, in order independent predictors of SBT success to be identified. High intercorrelated values were opted out for further analysis and so was f/Vt index, since all patients with f/Vt>105 were a priori grouped as having SBT failure. ΔScvO_2_% was calculated as ((ScvO_2_end-ScvO_2_start)/ScvO_2_start)*100. Its median value for the failure group was used as a potential threshold and multivariate analysis rerun with ΔScvO_2_ as a binary variable. A level of p<0.05 was considered significant to identify independent predictors of SBT success. Odds Ratios (OR) with corresponding 95% Confidence Intervals (CI) were reported for each predictor. Moreover, a Receiver Operating Characteristic (ROC) Curve was constructed for each SBT predictor, and the area under the curve was used as a visual index of the diagnostic accuracy of each one of them.

## 3 RESULTS

A total of 77 patients (69(18-86) years old; 62.3% male) constituted the study population (Table **1**). The main diagnosis of ICU entry were infections (such as pneumonia or abdominal infections) (32.5%), major surgery (chest, abdominal, brain, vascular or orthopedic surgery) (39%), cardiac arrest (3.9%), trauma (9.1%), acute respiratory failure (2.5%), status epilepticus (3.9%), burns (1.3%), stroke (3.9%) and intoxication (3.9%). Overall, 49 patients (63.6%) had a successful SBT, with the rest 28 (36.4%) having a SBT failure. Patients who had a successful SBT were younger (66 (25) vs. 73.5 (11) years old; p=0.024), while no other difference was noted between the groups in demographic and rest of baseline characteristics (Table **1**).

(Table **2**) presents all measurements which were contacted just before and/or at the end of the SBT. Apart from Hb concentration, which was significantly lower among those who failed the SBT (8.9 (1.9) vs. 9.8 (2.2) g/dl; p=0.028), compared to those who succeeded, no difference was noted between the two groups, regarding measurements which were contacted just before the trial. On the contrary, by the end of it, the group of patients with successful SBT had higher SaO_2_end (97.8 (2) vs. 97.5 (1) %; p=0.014), higher ScvO_2_end (77.2 ± 7.8 vs. 71.9 ± 8.4%; p=0.007), a trend towards higher PaO_2_end (104.9±32 vs. 91.3±27.7 mm Hg; p=0.063), lower O_2_ERend (20.9±7.7 vs. 25.4±7.9; p=0.020) and lower f/Vt index (60±26.37 vs. 106.2±48.3; p<0.001), compared to those with SBT failure.

**Fig. (1):**
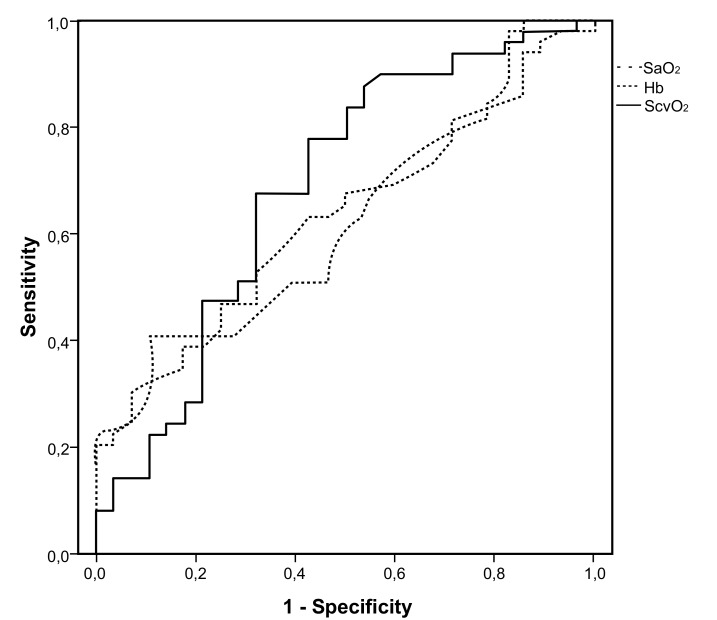
ROC curves for Hb concentration, SaO_2_ (at the end of the SBT) and ScvO_2_ (at the end of the SBT) for predicting a successful SBT outcome.

The final multivariate model included age, Hb, SaO_2_end and ScvO_2_end. O_2_ERend was opted out due to high intercorrelation to ScvO_2_end (r=0.982). SaO_2_end, Hb concentration and ScvO_2_end were positively associated, while age was negatively associated with SBT success (Table **3**). (Fig. **1**) presents ROC curves for ScvO_2_end, SaO_2_end and Hb concentration for a successful SBT outcome, with an area under curve being 0.685, 0.625 and 0.629 for ScvO_2_end, SaO_2_end and Hb concentration, correspondingly. Median ΔScvO_2_% value for the SBT failure group was equal to -4.2%, so we rerun the multivariate regression analysis including the binary parameter ΔScvO_2_% (≥4% or <4%) instead of ScvO_2_end. A decrease of less than 4% in ScvO_2_ values between the beginning and the end of an SBT was also independently associated with a successful outcome (OR=18.278; 95% CI=4.017-83.163, p<0.001), along with age, Hb concentration and SaO_2_end. The area under the curve for ΔScvO2% in predicting a successful SBT was 0.712

## 4 DISCUSSION

In this prospective, nested case-control study we indicated that ScvO_2_end is an independent predictor of SBT outcome, with a decrease of ScvO_2_ <4% being associated with SBT success. It is well-known that the characterization of a SBT as success or failure is normally based on a combination of subjective criteria and objective indices measured at the end of the 30 minute trial, according to guidelines, which do not include the evaluation of any ScvO_2_ values. However, our study confirms that ScvO_2_end as a single index is independently associated with a positive outcome and although its diagnostic accuracy was moderate (68.5% for ScvO_2_ and 71.5% for ΔScvO_2_), it was slightly superior to that of SaO_2_, which is a routinely screened parameter.

According to the Fick principle, oxygen uptake (VO_2_) depends on oxygen delivery (DO_2_) and O_2_ER [17, 18]. This classic equation could be reformulated as a function of SvO_2_, indicating that the four components which are associated with SvO_2_ values are Cardiac Output (CO), Hb concentration, VO_2_ values and SaO_2_ values; However, the relationship between SvO_2_ and its components are not equivalent and not necessarily linear [19]. In a previous study where the relationship between SvO_2_ and CO was modeled using a standard population of 1000 ICU patients, the curve between CO and SvO_2_ was non-linear and CO was an important but not the predominant component of SvO_2_, except when very low [19]. In any case, SvO_2_ seems to be a variable that passively follows the regulation of its components so that for any change in VO_2_ needs, the tissue oxygenation represents the final adjustment between O_2_ delivery and uptake and determines the change in SvO_2_ [19]. Previously Jubran * et al* [11] utilized SvO_2_ monitoring in order to assess cardiovascular performance and global tissue oxygenation during the weaning process; In this study the group of patients who failed weaning established a progressive decrease in SvO_2_ values, compared to the rest, probably reflecting the increased O_2_ER of respiratory muscles [11, 20]. According to Zakynthinos * et al* [12], in mechanically ventilated patients who failed to wean and presented with increased oxygen consumption, this was met by an increased tissue O_2_ER and was reflected by a drop in SvO_2_ values, confirming the association between SvO_2_ and tissue oxygen delivery and consumption.

Previous studies have assessed the utility of SvO_2_, ScvO_2_ or their changes during weaning procedures. Among ten postoperative coronary artery bypass graft patients, Cason * et al* indicated that SvO_2_ was an important predictor of weaning, since weaning failure was associated with a drop of SvO_2_ of over 60% [15]. However the study size was minimal and the patient population was selected. In another selected population of postoperative coronary artery bypass graft patients, Armaganidis and Dhainaut [21] demonstrated that SvO_2_ >60% was the best weaning success predictor and it depended on O_2_ER measurements. More recent studies concluded that change of ScvO_2_ during SBT was the only independent predictor of weaning from mechanical ventilation among patients who passed the first step of a two-step weaning procedure [16] and among difficult-to-wean patients [2]. In these studies, a drop of more than 4.5% [16] or 5% [2] in ScvO_2_ values, was associated with failure to wean from mechanical ventilation. Both protocols, though, investigated not whether ScvO_2_ is an index of a successful SBT, like in our study, but whether it is as a predictor of the extubation outcome within the following 48 hours. Nevertheless, laryngeal edema, low muscle endurance and inability to clear secretions are only some of the several potential causes of re-intubation, which are not directly associated to oxygen consumption and utilization, so these results may be, to an extent, difficult to explain.

In the current study, Hb was also independently associated with SBT outcome and had a similar diagnostic accuracy to ScvO_2_ values. In a large retrospective study conducted by Lai YC * et al*. [22]. Hb concentration was positively associated with a successful weaning, among difficult-to-wean patients, while in another smaller cohort lower Hb levels were associated with extubation failure [23], confirming our findings. The oxygen-carrying capacity of the blood depends directly on the level of Hb [24], and in normal individuals, 15 g/dl of Hb carry approximately 21 ml of oxygen per 100 ml of blood [25]. In these subjects, a 3 g/dl decrease in Hb levels would result in a reduction of the total oxygen-carrying capacity by 4/100 ml; this effect might be even more intense among patients with respiratory failure since Hb saturation is usually abnormal [26]. Under this scope, the reduction in oxygen-carrying capacity might further impede the aerobic metabolism of respiratory muscles, resulting in respiratory inefficiency and weaning failure.

SaO_2_ was also identified as an independent predictor of SBT success. This result comes to an agreement with previous data which indicated that an integrative index comprising SaO_2_ had a higher diagnostic accuracy in predicting the weaning outcome than other traditional indices [27]. When the arterial oxygen content is low, the diffusion gradient of oxygen from the blood to the mitochondria decreases more rapidly, potentially leading respiratory muscles to early anaerobic metabolism [26]. Surprisingly, the PaO_2_ end could not discriminate between the two groups in our study, neither at the beginning nor at the end of the trial, confirming the findings of similar studies in the field [2, 16]. Traditionally, the PaO_2_/FiO_2_ ratio has been used to identify patient populations ready to wean from mechanical ventilation [28-30]; Nevertheless, whether the diagnostic accuracy of PaO_2_ as a single index is similar to that of oxygen tension as part of composite indices remains to be investigated in the future.

The current study carries its strengths and limitations. Its prospective design minimized missing data and recall bias, while the inclusion of a general medical and surgical population allows for the generalizability of the results. Moreover, the same medical team evaluated readiness to wean and the SBT outcome, using similar criteria, so methodological bias, such as the ones that could be encountered in multi-center studies have been minimized. Although the sample size is relatively small, it is comparable to other similar studies in the field [2, 23]. A limitation of the study is that several weaning indices, such as airway occlusion pressure (P0.1), the P0.1/MIP ratio [27], and the integrative weaning index (static compliance of the respiratory system-arterial oxygen saturation/f/vt index) [31] have not been tested; However, the aim of the study was to investigate whether the diagnostic accuracy of ScvO_2_ in predicting the SBT outcome was similar to the one of other parameters which are routinely used for patient assessment, according to guidelines. Another limitation is that no hemodynamic measurements to assess CO were conducted in the patient cohort, nor we obtained any measurements of SvO_2_, due to the limited use of right heart catheterization; However, previous studies [32-34] demonstrated adequate correlation between ScvO_2_ and SvO_2_, especially among patients who are not in shock, while the costs and the potential risks of right heart catheterization make this technique less feasible in every-day clinical practice. Furthermore, most of the impact of CO on SvO2 measurements is evident when CO values are low [19]; Since none of the patients was hemodynamically unstable when SBT was attempted, we could hypothesize that measuring CO would not have changed the results. Finally, VO_2_ was not directly measured and, thus, increase of VO_2_ due to elevated respiratory muscle activity among patients who presented with lower ScvO_2_ can only be hypothesized; future studies utilizing indirect calorimetry through a mouthpiece could provide further information on this proposed association.

In summary, in this prospective nested case-control study conducted in a general ICU population, a ScvO_2_ drop of at least 4% was independently associated with failure to wean from mechanical ventilation, along with Hb concentration, SaO_2,_ and age. Judging whether the SBT is successful or not, in order to proceed to extubation, is depended not on a single index but on a combination of several objectively measured and clinically evaluated parameters. Whether adding the routine evaluation of ScvO_2_ in this algorithm might improve the ability to correctly identify those patients who could safely proceed to extubation or not, remains to be tested in large prospective trials in the future.

## CONCLUSION

ScvO_2_ is an independent predictor of the weaning outcome; a drop of at least 4% from its baseline values was associated with failure to wean from mechanical ventilation. More prospective studies are needed in order to investigate whether its systematic evaluation might further facilitate the accurate identification of those able to wean from mechanical ventilation.

## Figures and Tables

**Table 1:** Baseline characteristics of the study population.

**Variable**	**Total population (N=77)**	**SBT success** **(N1=49)**	**SBT failure** **(N2=29)**	**p**
**Age, yrs**	69 (24)	66 (25)	73.5(11)	0.024
**Gender % (n)** -male -female	62.3 (48) 37.7 (29)	70.8 (34) 51.7 (15)	29.2 (14) 48.3 (14)	0.091
**APACHE II score**	19.4±7	19.1±7.3	19.9±6.7	0.639
**CVC placement %, (n)** **-**jugular -subclavian -femoral	54.5 (42) 9.1 (7) 36.4 (28)	64.3 (27) 57.1 (4) 64.3 (18)	35.7 (15) 42.9 (3) 35.7 (10)	0.932
**Days on mechanical ventilation until first SBT**	4 (4)	4 (4)	4 (4)	0.542

SBT: Spontaneous breathing trial

CVC: central venous catheter

**Table 2:** Parameters which were evaluated in the two groups just before and at the end of SBT.

**Variable**	**Total population (N=77)**	**SBT success** **(N1=49)**	**SBT failure** **(N2=29)**	**p**
*Hb, g/dl	9.5 (2.4)	9.8 (2.2)	8.9 (1.9)	0.028
*FiO_2_	0.4 (0.1)	0.4 (0.1)	0.4 (0.1)	0.958
*PaO_2_/FiO_2_	273.3 (145.3)	280.1±95.7	292.4±90.1	0.601
*MIP, cm H_2_0	40 (24)	40 (22)	40 (32)	0.395
SaO_2_ start, %	98 (1.5)	98 (1.4)	98 (1.7)	0.675
SaO_2_ end, %	97.7 (2)	97.8 (2)	97.5 (1)	0.014
ScvO_2_ start, %	75.6±8	76 ± 8.8	74.9 ± 6.6	0.573
ScvO_2_ end, %	75.3±8.4	77.2 ± 7.8	71.9 ± 8.4	0.007
PaO_2_ start, mm Hg	109.4±27.7	108.4±27.2	111.1±29	0.958
PaO_2_ end, mm Hg	98±31	104.9±32	91.3±27.7	0.063
pH start	7.43±0.04	7.42±0.05	7.43±0.04	0.680
pH end	7.42±0.04	7.42±0.04	7.42±0.04	0.840
HCO_3_ start	26.6±5.4	26.6±5.5	26.5±5.4	0.967
HCO_3_ end	27.1±5.9	26.8±6.1	27.5±5.6	0.592
PaCO_2_ start, mm Hg	40.9±9.5	41.2±10.2	40.5±8.5	0.744
PaCO_2_ end, mm Hg	42.3±11.2	41.9±11.9	43±10.1	0.690
O_2_ER start	22.7±8	22.3±8.8	23.3±6.6	0.611
O_2_ER end	22.5±8	20.9±7.7	25.4±7.9	0.020
HR start, beats/min	93.8±15.3	95.1±14.6	91.4±16.5	0.307
HR end, beats/min	99.4±16.1	99.4±15.2	99.5±17.8	0.968
MAP start, mm Hg	93.3±13.1	95.1±13.2	90.1±12.6	0.107
MAP end, mm Hg	95.1±14.7	96.9±14.9	91.8±13.9	0.136
^#^f/Vt	77.6±42.7	60±26.37	106.2±48.3	<0.001

SBT: Spontaneous breathing Trial; Hb: hemoglobin concentration; FiO_2_: Fraction of Inspired Oxygen; PaO_2_: arterial oxygen partial pressure; MIP: maximum inspiratory pressure; Start: measured just before the beginning of the SBT; End: measured at the end of SBT; SaO_2_: arterial oxygen saturation; ScvO_2_: central venous oxygen saturation; HCO3: bicarbonate anion; PaCO_2_: arterial carbon dioxide partial pressure; O_2_ER: Oxygen extraction ratio; HR: heart rate; MAP: mean systemic arterial blood pressure

* Measured only once, just before SBT initiation

^#^ Measured only once, at the end of SBT

**Table 3:** Multivariate predictors of SBT successful outcome.

**Variable**	**OR**	**95% CI**	**p**
ScvO_2_ end(%)	1.122	1.005-1.257	0.041
SaO_2_ end(%)	1.405	1.041-1.897	0.026
Age (y)	0.961	0.923-0.999	0.049
Hb (g/dl)	1.549	1.080-2.221	0.017

Hb: hemoglobin concentration; SaO_2_: arterial oxygen saturation; ScvO_2_: central venous oxygen saturation

## LIST OF ABBREVIATIONS IN ALPHABETICAL ORDER

APACHE: = Acute Physiology and Chronic Health Evaluation
Ca: = Calcium
CI: = Confidence Interval
CO: = Cardiac Output
DBP: = Diastolic Blood Pressure
DO_2_: = Oxygen Delivery
f: = Respiratory Frequency
FiO_2_: = Fraction of Inspired Oxygen
Hb: = Hemoglobin Concentration
HCO3: = Bicarbonate Anion
HR: = Heart Rate
ICU: = Intensive Care Unit
K: = Potassium
MIP: = Maximum Inspiratory Pressure
Na: = Sodium
OR: = Odds Ratio
O_2_ER: = Oxygen Extraction Ratio
PEEP: = Positive End Expiratory Pressure
RSBI: = Rapid Swallow Breathing Index
P0.1: = Airway Occlusion Pressure
PaCO2: = Arterial Carbon Dioxide Partial Pressure
PaO_2_: = Arterial Oxygen Partial Pressure
ROC: = Receiver Operating Characteristic
SaO_2_: = Arterial Oxygen Saturation
SBP: = Systemic Systolic Blood Pressure
SBT: = Spontaneous Breathing Trial
ScvO_2_: = Central Venous Oxygen Saturation
SvO_2_: = Mixed Venous Oxygen Saturation
VO_2_: = Oxygen Uptake
Vt: = Tidal Volume
ΔScvO2: = Change in ScvO_2_ values between the beginning and the end of SBT
